# Changes in RANKL, OPG, and 25(OH)D Levels in Children with Leukemia from Diagnosis to Remission

**DOI:** 10.3390/cancers16162811

**Published:** 2024-08-10

**Authors:** Salvador Atilano-Miguel, Lourdes Barbosa-Cortés, Rocío Ortiz-Muñiz, Jorge Maldonado-Hernández, Jorge A. Martin-Trejo, Maricela Rodríguez-Cruz, Lourdes Balcázar-Hernández, Karina A. Solís-Labastida, Benito A. Bautista-Martínez, Azalia Juárez-Moya, Zayra Hernández-Piñón, Raeline A. Galindo-Rodríguez, Adriana Chávez-Anaya, Rosa E. Valdez-Avilez, Juan M. Domínguez-Salgado, Judith Villa-Morales, María E. Rodríguez-Palacios

**Affiliations:** 1 Doctorado en Ciencias Biológicas y de la Salud, Universidad Autónoma Metropolitana, Ciudad de México 14387, Mexico; atilanosalvador@gmail.com (S.A.-M.); arom@xanum.uam.mx (R.O.-M.); 2 Unidad de Investigación Médica en Nutrición, Unidad Médica de Alta Especialidad (UMAE), Instituto Mexicano del Seguro Social (IMSS), Hospital de Pediatría, Centro Médico Nacional Siglo XXI, Ciudad de México 06720, Mexico; jormh@yahoo.com.mx (J.M.-H.); maricela.rodriguez.cruz@gmail.com (M.R.-C.); raeline.2405@gmail.com (R.A.G.-R.); adriichavez03@hotmail.com (A.C.-A.); jmdomin6@outlook.com (J.M.D.-S.); jusyvm@hotmail.com (J.V.-M.); mrodriguezp2401@alumno.ipn.mx (M.E.R.-P.); 3 Departamento de Ciencias de la Salud, División de Ciencias Biológicas y de la Salud, Universidad Autónoma Metropolitana, Unidad Iztapalapa, Ciudad de México 9340, Mexico; rosa_5elena@hotmail.com; 4 Departamento Clínico de Hematología, Unidad Médica de Alta Especialidad (UMAE), Instituto Mexicano del Seguro Social (IMSS), Hospital de Pediatría, Centro Médico Nacional Siglo XXI, Ciudad de México 06720, Mexico; jorge.martintr@imss.gob.mx (J.A.M.-T.); kas_anastacia@yahoo.com (K.A.S.-L.); bbautistamartinez@yahoo.com (B.A.B.-M.); dra.ajuarez@gmail.com (A.J.-M.); zayra_hp@hotmail.com (Z.H.-P.); 5 Departamento de Endocrinología, Unidad Médica de Alta Especialidad (UMAE), Instituto Mexicano del Seguro Social (IMSS), Hospital de Especialidades, Centro Médico Nacional Siglo XXI, Ciudad de México 06720, Mexico; ludab@comunidad.unam.mx

**Keywords:** acute lymphoblastic leukemia, RANK ligand, osteoprotegerin, 25(OH)D, corticosteroids, bone turnover markers

## Abstract

**Simple Summary:**

Advances in the treatment of acute lymphoblastic leukemia (ALL) have led to a marked improvement in the survival rate of patients. Nevertheless, these patients may develop adverse effects during and after treatment, such as bone abnormalities and vitamin D deficiency. Bone remodeling allows for bone volume and structure to be maintained, which is controlled by the receptor activator of the nuclear factor-kB (RANK)/RANK ligand (RANKL)/osteoprotegerin (OPG) system-determining pathway in the balance between bone formation and resorption. Some reports have explored the role of corticosteroids in modulating the RANKL and OPG levels and RANKL/OPG ratio in pediatric patients. Nevertheless, studies evaluating the role of RANKL and OPG in the bone health of pediatric ALL patients during treatment are limited. During remission, we observed an increase in the RANKL/OPG ratio, increased RANKL levels, and decreased OPG levels in ALL patients. These changes may predispose such patients to the development of bone health disorders in their adult lives.

**Abstract:**

Background: The receptor activator of the nuclear factor-kB (RANK)/RANK ligand (RANKL)/osteoprotegerin (OPG) pathway is a determining pathway in the balance between bone formation and resorption, and disruptions in this complex can affect bone metabolism. Methods: This study analyzes the changes in RANKL, OPG, and 25(OH)D levels; the RANKL/OPG ratio; and other bone turnover markers (BTMs) from diagnosis to complete remission in children with acute lymphoblastic leukemia (ALL). This is a prospective observational cohort study, carried out at the Instituto Mexicano del Seguro Social, Mexico City, including 33 patients (4–17 years) with newly diagnosed B-cell ALL. The patients were treated with the HP09 chemotherapy protocol. Children who had previously been treated with corticosteroids were excluded. A peripheral blood sample at diagnosis and remission was collected to determine the 25(OH)D and BTM concentrations. Results: Increased RANKL (*p* = 0.001) and osteocalcin (*p* < 0.001) levels and RANKL/OPG ratio (<0.001) and a decreased OPG level (*p* = 0.005) were observed at remission, predominantly in the high-risk (HR) relapse and vitamin D deficiency groups. A negative association between RANKL and OPG (r = −0.454, *p* = 0.008) was observed. Conclusions: we suggest that the RANKL/OPG ratio could serve as a bone remodeling marker in ALL patients.

## 1. Introduction

Advances in the treatment of acute lymphoblastic leukemia (ALL) have led to a marked improvement in the survival rate of patients, ranging from almost fatal to curable in 85–90% of patients [1]. Nevertheless, ALL patients may develop adverse effects during and after treatment [2,3,4], including decreased bone mineral density (BMD), osteopenia/osteoporosis, osteonecrosis, fragility fractures, and vitamin D deficiency [5,6,7,8,9].

Childhood and adolescence are critical stages for bone development, mineralization, and the attainment of peak bone mass [10]. Bone remodeling is a tightly regulated process that involves the repair of microdamage and replacement of old bone with new bone through osteoclastic resorption and osteoblastic bone formation [11]. The RANK/RANKL/OPG pathway controls osteoclastogenesis [12] and bone resorption [13]. The RANK/RANKL interaction induces the formation of multinucleated mature osteoclasts, leading to bone resorption, while the binding of OPG to RANKL inhibits osteoclastogenesis [14,15], and disruptions in this complex result in excessive or impaired bone remodeling [13].

Corticosteroids are among the most potent osteotoxic drugs that are routinely prescribed to treat serious childhood illnesses, including leukemia [16]. Corticosteroids affect bone formation by decreasing the number and differentiation of osteoblastic lineage cells, as well as stimulating osteoclast differentiation and function through increased RANKL production, decreased OPG production by osteoblasts, an increase in the RANKL/OPG ratio, and excessive osteocyte apoptosis, leading to increased bone resorption and increased fracture risk [17,18]. Additionally, low vitamin D levels are associated with increases in RANKL expression and the RANKL/OPG ratio [19]. Childhood ALL patients are at risk of impaired vitamin D status and bone metabolism, which could be caused by limited sun exposure, nutritional factors [20], decreased physical activity, corticosteroids, and methotrexate therapy [21,22]. In vivo studies have shown that corticosteroid administration downregulates the expression of the CYP24A7 and CYP27B1 mRNAs, both of which are essential for controlling the availability of the active metabolite of vitamin D, 1,25(HO)2D [23,24].

Some reports have explored the role of corticosteroids in modulating RANKL and OPG levels and the RANKL/OPG ratio in pediatric patients with chronic disorders, immobility, and corticosteroid exposure, as well as its relationship with bone health [19,25,26]. Nevertheless, studies evaluating the role of RANKL and OPG in bone health in pediatric patients during and after treatment are limited [9,27]. Muggeo et al. assessed the levels of bone turnover markers in ALL patients after the intensification phase and reported higher levels of RANKL and OPG in the ALL group than in the control group [9]. Hablas et al. reported higher concentrations of RANKL in ALL survivors than in healthy patients. Additionally, vitamin D levels were positively correlated with OPG and negatively correlated with RANKL levels [27]. In this context, the exploration of vitamin D [25(OH)D] levels and the impact of the administration of corticoids on the RANKL/OPG ratio in pediatric patients with ALL is relevant, as an imbalance in the expression or function of any component of this system can induce deregulation of the remodeling cycle and generate modifications in BMD. These effects can increase the long-term risk of osteoporosis and fractures in pediatric patients with ALL.

Therefore, our aim was to analyze the changes in RANKL, OPG, and 25(OH)D levels; the RANKL/OPG ratio; and other bone turnover markers (BTM) [osteocalcin, tartrate-resistant acid phosphatase type 5b (TRACP-5b), and bone alkaline phosphatase (BAP)] from diagnosis to complete remission in children with ALL.

## 2. Materials and Methods

### 2.1. Study Design and Participants

**Figure 1:**
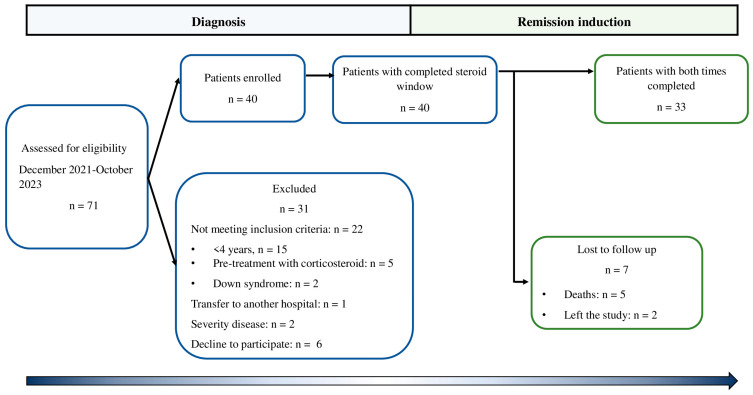
Study flow-chart in patients with ALL.

This prospective observational cohort study (from diagnosis to complete remission) was carried out in the Medical Nutrition Research Unit and the Clinical Department of Hematology of the Pediatric Hospital of the Institute Mexicano del Seguro Social (IMSS) in Mexico City, Mexico. The eligibility criterion was patients who were newly diagnosed with ALL before the beginning of chemotherapy treatment between December 2021 and November 2023. Children who had previously been treated with chemotherapy at another institution, who had Down syndrome, or who had previously been treated with corticosteroids were excluded. A total of 71 patients were newly diagnosed with B-cell ALL, of which 22 patients were excluded as they did not meet the inclusion criteria, while 2 others were excluded due to the severity of the disease. One patient was transferred to another hospital, and in six patients the legal representative did not authorize their participation in this study. Thus, 40 boys were enrolled in this study; however, five patients died from complications inherent to treatment, and two patients left the study. Therefore, 33 patients (4–17 years old) were included in the analysis. Patients were followed from the time of diagnosis of leukemia to remission (Figure 1). The patients were treated with the HP09 chemotherapy protocol based on the BFM 95 protocol [28]. The patients were stratified according to their risk of relapse as follows: standard risk (SR; >1 year to <7 years, initial leucocyte count of 20,000/mm^3^), intermediate risk (IR; >7 years to <10 years, initial leucocyte count of >20,000/mm^3^ and <50,000/mm^3^), and high risk (HR; <1 year and >10 years, initial leucocyte count of >50,000/mm^3^). Patients received monotherapy during the first seven days (50 mg/m^2^/d prednisone), multiple drugs during remission induction (29–33 days), prednisone 60 mg/m^2^/d for 28 days, and vincristine 1.5 mg/m^2^/d (4 doses), daunorubicin 30 mg/m^2^/d (2, 3, or 4 doses at standard, intermediate, and high risk, respectively), L-asparaginase 5000 IU/m^2^/d (8 doses to standard and intermediate, 6 doses to high risk), and 3 doses of intrathecal chemotherapy. According to the HP09 chemotherapy protocol, the complete remission (less than 5% blast) of the patient was monitored at 33 days by bone marrow aspirate. Recruitment, follow-up, and remission of the patients were at different times. Patients reached remission at 39 (32–39) days in the standard and intermediate risk group (SI-risk) and 38 (34–43) days in the high-risk (H-risk) group.

### 2.2. Clinical Data

The demographic and clinical characteristics of the patients were collected during recruitment (diagnosis) and follow-up (at complete remission). Body weight (kg) and body composition were measured by impedance using an InBody 230 (InBody USA, Cerritos, CA, USA) while the patients were wearing lightweight clothing. Height was measured with a wall-mounted stadimeter (Seca 222, Seca Corp., Oakland Center, Columbia, MD, USA). Body mass index (BMI) was calculated as weight (kg) divided by the square of the height (m); BMI scores were obtained from the World Health Organization (WHO) normative curves [29].

### 2.3. Analytical Methods

#### 2.3.1. Blood Samples

Peripheral blood samples at baseline, just before starting chemotherapy (diagnostic time), and during complete remission in the absence of relapse were collected between 8:00 and 9:00 am after an 8–12 h overnight fast. Clotted blood samples were centrifuged for 15 min at 3000 rpm under cold conditions (4 °C). Aliquots of serum and plasma were immediately frozen (−80 °C) and used to determine the serum concentrations of 25(OH)D and the plasma concentrations of RANKL, OPG, osteocalcin, BAP, and TRACP-5b. According to the HP09 chemotherapy protocol based on the BFM 95, complete remission (less than 5% blast) of the patient was monitored at 33 days according to bone marrow aspirate.

#### 2.3.2. Biochemical Assays

Serum concentrations of 25(OH)D were determined through a chemiluminescent microparticle immunoassay using a kit from Abbott (Abbott Park, IL, USA). The 25(OH)D concentrations were classified according to the Endocrine Society as follows: vitamin D deficiency was defined as 25(OH)D < 20 ng/mL, vitamin D insufficiency was defined as 25(OH)D of 21–29 ng/mL, and sufficiency was defined as 25(OH)D ≥ 30 ng/mL [30].

The concentrations of RANKL were determined using a Human RANKL Magnetic Bead assay (HRNKLMAG-51K-01; Merck KGaA, Darmstadt, Germany) with an analytical sensitivity of 0.5 pg/mL and a range of 4.88–20,000 pg/mL. OPG and osteocalcin concentrations were determined using a Human Bone Magnetic Bead Panel (HBNMAG-51K; Merck KGaA, Darmstadt, Germany) with an analytical sensitivity of 1.9 pg/mL and a range of 7–30,000 pg/mL for OPG and an analytical sensitivity of 68.5 pg/mL and a range of 146–600,000 for osteocalcin. The RANKL/OPG ratio was calculated for each patient through dividing the value of RANKL by that of OPG.

In a subsample of 20 patients, we analyzed BAP and TRACP-5b levels through ELISA. BAP was measured using a commercial kit (MBS60806; MyBiosource Inc., San Diego, CA, USA) with an analytical sensitivity of 1.89 IU/L and a range of 3–900 IU/L, where high levels were defined as ≥75th percentile [31]. TRACP-5b was assessed using a commercial kit (MBS045195; MyBiosource Inc., San Diego, CA, USA) with an analytical sensitivity of 0.1 IU/L and a range of 0.5–16 IU/L.

### 2.4. Statistical Analysis

Statistical analysis was performed using the SPSS Statistics version 23.0 software (IBM, Armonk, NY, USA). The data were analyzed according to total sample size (n = 33), vitamin D deficiency/insufficiency group, and risk of ALL relapse from diagnosis to complete remission. The data distribution was assessed with the Shapiro‒Wilk test. The quantitative data are presented as the means ± standard deviations (SDs) for normally distributed data or as the medians (minimal, maximal). Categorical variables are presented as percentages. To analyze changes in 25(OH)D levels, the RANKL/OPG ratio, and the levels of MBT during the follow-up, we used a Wilcoxon test or paired Student’s *t*-test according to the data distribution. The differences between groups by relapse risk were evaluated by Student’s *t*-test or the Mann–Whitney U-test. To examine the associations between 25(OH)D and corticosteroid dose and the RANKL/OPG ratio, Spearman’s correlation analysis was performed. The sample size had a value of α = 0.05, β = 0.1, and a statistical power of 1 − β = 0.9. Statistical significance was defined as *p* < 0.05.

## 3. Results

The demographic, anthropometric, body composition, clinical parameters, and BTM data of the ALL patients at diagnosis are presented in Table 1.

### 3.1. Changes in Bone Turnover Markers and the RANKL/OPG Ratio

**Figure 2:**
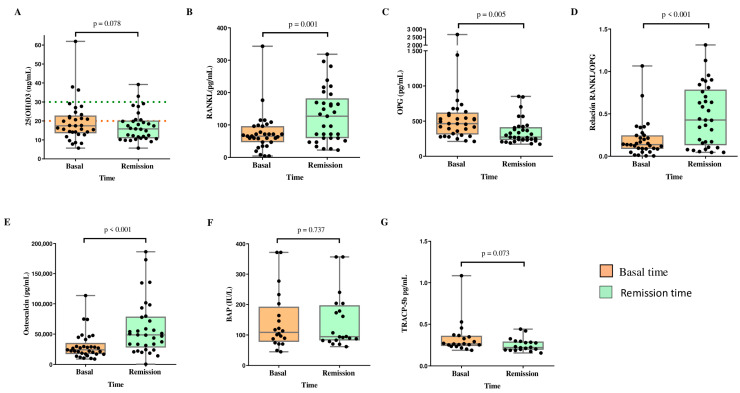
Changes in 25(OH)D, BMT, and RANKL/OPG ratio between diagnosis (baseline) and remission time in patients with ALL. Significant differences between the two time points were determined by Wilcoxon signed rank test. (**A**) 25(OH)D; (**B**) RANKL, receptor activator for nuclear factor kB ligand; (**C**) OPG, osteoprotegerin; (**D**) RANKL/OPG ratio; (**E**) osteocalcin; (**F**) BAP, bone alkaline phosphatase; (**G**) TRACP-5b, tartrate-resistant acid phosphatase. BAP and TRACP-5b, sample size analyzed n = 20. Sufficiency 25(OH)D ≥ 30 ng/mL (dotted green), and deficiency 25(OH)D < 20 ng/mL (dotted red).

Figure 2 shows the changes in 25(OH)D and BTM concentrations in the study population. The 25(OH)D levels tended to decrease between baseline and remission. We observed an increase in the serum concentrations of RANKL (65.5 pg/mL vs. 127.6 pg/mL, *p* = 0.001) and osteocalcin (24,098 pg/mL vs. 48,789 pg/mL, *p* < 0.001), as well as an increase in the RANKL/OPG ratio (0.137 vs. 0.427, *p* < 0.001) between baseline and remission. OPG concentrations were decreased at remission compared with basal levels (463.9 pg/mL vs. 273.0 pg/mL, *p* = 0.005). Moreover, the TRACP-5b concentration tended to decrease at remission compared with baseline (*p* = 0.073). BAP did not change during the follow-up.

### 3.2. Changes in Bone Turnover Markers and the RANKL/OPG Ratio According to the Risk of Relapse

Comparisons of 25(OH)D and BTMs levels between baseline and remission according to the risk of relapse in SI-R vs. HR were performed. In the SI-R group, we observed increased osteocalcin concentrations (29,227.2 pg/mL vs. 49,993.5 pg/mL, *p* = 0.01) between baseline and remission, with a tendency toward increased RANKL levels and RANKL/OPG ratio and decreased 25(OH)D and OPG concentrations at remission. However, we did not observe changes in BAP or TRACP-5b concentrations.

**Figure 3:**
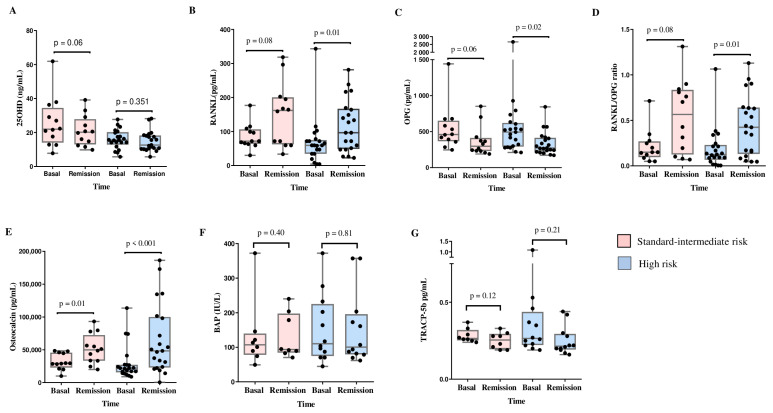
Changes in 25(OH)D3, bone turnover markers, and RANKL/OPG ratio between diagnosis (baseline) and remission time by risk of relapse in patients with ALL. Significant differences between the two time points were determined by Wilcoxon signed rank test. (**A**) 25(OH)D; (**B**) RANKL, receptor activator for nuclear factor kB ligand; (**C**) OPG, osteoprotegerin; (**D**) RANKL/OPG ratio; (**E**) osteocalcin; (**F**) BAP, bone alkaline phosphatase; (**G**) TRACP-5b, tartrate-resistant acid phosphatase. Standard or intermediate risk of relapse (n = 12) and high risk of relapse (n = 21). BAP and TRACP-5b sample size analyzed n = 20. Sufficiency 25(OH)D ≥ 30 ng/mL (dotted green), and deficiency 25(OH)D < 20 ng/mL (dotted red).

The HR group presented increased levels of RANKL (59.3 ng/mL vs. 96.3 ng/mL, *p* = 0.01), osteocalcin (22,090 pg/mL vs. 48,739 pg/mL, *p* < 0.001), and RANKL/OPG ratio (0.125 vs. 0.424, *p* = 0.01); moreover, the OPG level (493.5 pg/mL vs. 272.0 pg/mL, *p* = 0.02) was decreased at remission compared with baseline. There were no changes in the 25(OH)D, BAP, or TRACP-5b concentrations (Figure 3).

As expected, a difference in the cumulative dose of prednisone between the SI-R and HR groups was observed (1906.1 ± 460.4 mg/m^2^ vs. 2972.6 ± 1003.3 mg/m^2^, respectively; *p* = 0.002).

### 3.3. Changes in Biochemical Bone Turnover Markers and the RANKL/OPG Ratio According to Vitamin D Status

**Figure 4:**
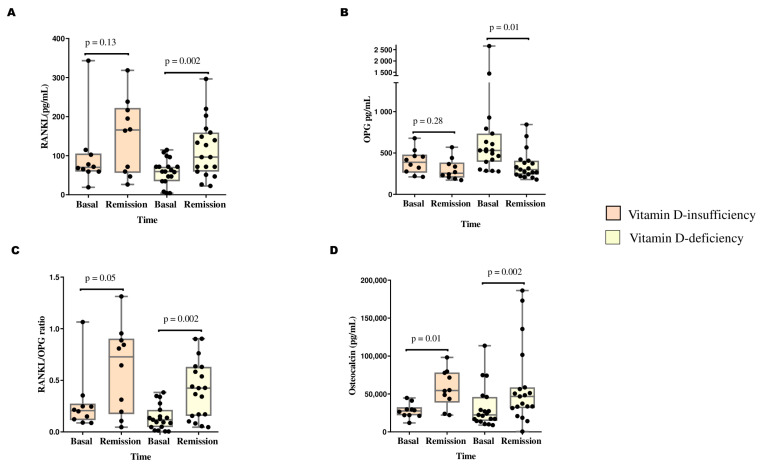
Changes in bone turnover markers and RANKL/OPG ratio between diagnosis (baseline) and remission time according to vitamin D status in patients with ALL. Significant differences between the two time points were determined by Wilcoxon signed rank test. (**A**) RANKL, receptor activator for nuclear factor kB ligand; (**B**) OPG, osteoprotegerin; (**C**) RANKL/OPG ratio; (**D**) osteocalcin. Insufficient vitamin D (n = 10) levels, deficient vitamin D levels (n = 20).

BTMs and the RANKL/OPG ratio were compared between baseline and at remission according to vitamin D deficiency status (Figure 4). The vitamin D-deficient group presented increases in RANKL (54.3 pg/mL vs. 96.3 pg/mL, *p* = 0.002), osteocalcin (22,413 pg/mL vs. 46,816 pg/mL, *p* = 0.002), and the RANKL/OPG ratio (0.118 vs. 0.424, *p* = 0.002) and decreased OPG (530.6 pg/mL vs. 299.0 pg/mL, *p* = 0.01). Moreover, the vitamin D-insufficient group presented increases in osteocalcin levels (27,575.5 pg/mL vs. 57,215.0 pg/mL, *p* = 0.01) and the RANKL/OPG ratio (0.207 vs. 0.727, *p* = 0.05)—see Figure 4.

### 3.4. Associations among Bone Turnover Markers, 25(OH)D Concentration, and Corticosteroid Dose

**Figure 5:**
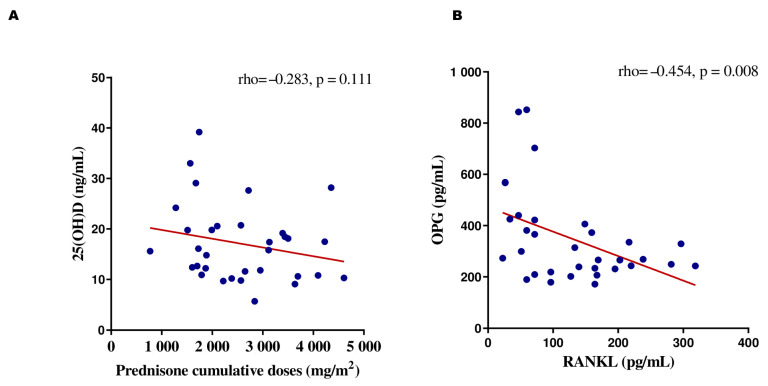
Spearman’s correlations between (**A**) vitamin D concentrations and cumulative corticosteroid doses and (**B**) OPG and RANKL in patients with ALL at remission time.

The correlations among the BTM concentration, 25(OH)D concentration, and corticosteroid dose were analyzed during remission. A negative correlation was observed between RANKL and OPG plasma levels (r = −0.454, *p* = 0.008). The dose of prednisone did not correlate with the serum 25(OH)D, RANKL, OPG, osteocalcin, BAP, or TRACP-5b concentrations or the RANKL/OPG ratio (r = −0.283, r = 0.183, r = −0.240, r = 0.178, r = 0.213, r = −0.283, and r = 0.182, respectively; *p* ≥ 0.99; Figure 5).

## 4. Discussion

In this report, we present an analysis of changes in RANKL and OPG levels and the RANKL/OPG ratio in children with ALL from diagnosis to remission without any intervention in addition to hematological treatment. In this study, we observed increases in RANKL levels, osteocalcin levels, and the RANKL/OPG ratio, as well as a decrease in OPG levels at remission. When relapse risk and vitamin D were considered, patients with an HR of relapse or vitamin D deficiency presented these changes, which may reflect accelerated bone remodeling.

The RANKL/RANK/OPG axis plays an essential role in the regulation of bone metabolism, especially in bone remodeling. This lifelong process is characterized by an equilibrium between the activities of osteoclasts and osteoblasts [12]. Several studies have evaluated RANKL and OPG levels and the RANKL/OPG ratio in different pediatric diseases [19,25,26,32,33], but few studies have evaluated changes in bone health parameters in patients with ALL during treatment and without any intervention. In this sense, Solmanz et al. [34] have evaluated the effect of a combination of vitamin K2 (menaquinone-7 100 µg/day) and vitamin D3 (calcitriol 10 µg/day) on BMD, RANKL, and OPG levels, among other biochemical markers of bone turnover, in 29 patients with ALL (1.0–17 years) who were randomized into a study group (n = 15) and a control group (n = 14). Patients were evaluated from diagnosis to the first, second, third, and sixth months of treatment in this study. Nevertheless, they did not find any differences in RANKL or OPG concentrations between groups or within groups during the follow-up. The OPG/RANKL ratio differed between groups in the first month. In contrast, even though we had a shorter follow-up time than Solmaz et al. [34], and without any intervention with vitamin D, we observed an increase in the RANKL concentration and the RANKL/OPG ratio, as well as a decrease in OPG level between diagnosis and remission; these results suggest that, during the early phase of ALL treatment, glucocorticoid administration mainly increased osteoclastic activity and, consequently, bone resorption in these patients. Similarly, we observed the same changes in the HR and vitamin D deficiency groups. This observation could be explained, in part, by the higher cumulative glucocorticoid dose in the HR group than in the SI-R group; consequently, this group presented increased RANKL levels and decreased OPG levels during follow-up [18]. In addition, low vitamin D levels are associated with increased RANKL [27], which could explain the increase in RANKL in the vitamin D-deficient group of patients. We know that glucocorticoids are some of the most potent osteotoxic drugs that are routinely prescribed to treat serious childhood illnesses such as leukemia. The reduction in bone mass by glucocorticoids involves systemic and direct effects on bone cells, leading to the induction of apoptosis in osteoblasts and osteocytes, as well as suppression of their differentiation, which can be explained by a mechanism involving the suppression of cytokines such as interleukin 11 and activator protein 1 (AP-1) [35]. On the other hand, glucocorticoids increase the proliferation and differentiation of osteoclastic precursors by synthesizing receptor RANKL, an essential stimulator of osteoclastogenesis. Humphrey et al. have reported that dexamethasone, prednisolone, and deflazacort inhibited OPG production and stimulated RANKL mRNA in two human osteoblastic cell lines, where these changes were accentuated with increasing dose, and these cellular effects of glucocorticoids are mediated by the glucocorticoid receptor [36]. The glucocorticoid receptor causes an increase in RANKL promoter activity and increased RANKL mRNA expression level [37], while OPG could be inhibited through AP-1 and the suppression of b-catenin signaling by glucocorticoids [38].

According to the 25(OH)D serum levels, there was a high prevalence of hypovitaminosis D both at diagnosis (~90%) and at remission (94%). Remarkably, this percentage was higher than that reported by ENSANUT 2018 for healthy Mexican preschoolers and schoolchildren (73.2% and 75%, respectively) [39]. These results are consistent with those reported previously in several studies on patients with ALL receiving early-phase treatment [21,40,41]. In contrast to studies that have reported a decrease in 25(OH)D levels during the treatment of children with ALL, we only found a trend toward a decrease in the level of 25(OH)D during remission.

In addition, we observed a negative correlation between RANKL and OPG plasma levels, which could explain the increase in the RANKL/OPG ratio. This is important, as a high RANKL/OPG ratio has been associated with increased bone resorption [42]. Additionally, the increase in RANKL may be associated with an initial increase in the signaling and activity of osteoclasts, which could lead to a predisposition to osteopenia and osteoporosis [33]. We hypothesized that the increase in both molecules could result in bone abnormalities, such as decreased bone mineral density; however, more research is required to further understand the impact on healthy bone.

TRAP-5b is an enzyme that is highly expressed in osteoclasts and is a regulator of bone resorption [43]. In pediatric patients with ALL, circulating TRACP-5b concentrations are reportedly greater than those in healthy individuals [9]. Solmaz et al. [34] did not observe changes during the 6 months of follow-up. Although we observed a significant increase in RANKL levels at remission, we found a tendency toward a decrease in TRACP-5b levels only between diagnosis and remission. A decrease in TRACP-5b has been reported in patients with myeloma who underwent induction treatment with vincristine–doxorubicin–dexamethasone–pamidronate [44]. In particular, long-term glucocorticoid treatment reduces TRACP-5b concentrations. These results could be partly explained by disruptions in the cytoskeleton of osteoclasts generated by long-term glucocorticoids that reduce their activity [45].

Moreover, BAP and osteocalcin are considered markers of bone formation. BAP is an isoenzyme of alkaline phosphatase which is produced from bone and is an indicator of osteoblastic activity, while osteocalcin is a protein synthesized by mature osteoblasts that plays an important role in metabolic regulation, bone mineralization, and calcium homeostasis. Orgel et al. [46] have evaluated bone structure, density, and circulating BTM at diagnosis and changes during induction therapy in pre-adolescents, adolescents, and young adults (n = 38) with ALL and sex-matched healthy controls (n = 38). The authors reported a decrease in bone structure and BMD. However, there were no changes in BAP or osteocalcin levels during follow-up, while the levels of C-telopeptide—a resorption marker—were increased. Our BAP results are consistent with those of Orgel et al.; in contrast, we reported increased osteocalcin levels in pediatric patients with ALL in complete remission. This could be explained by the increase in RANKL levels, as RANKL is a central player in osteoclast activation and bone destruction [47]. Osteocalcin can bind to the bone matrix, as it contains three glutamate residues that can be carboxylated; this modification allows it to bind to calcium and hydroxyapatite. Hence, the resorption of the bone matrix by osteoclasts can result in the release and decarboxylation of bound carboxylated osteocalcin, which could increase osteocalcin levels [48,49]. A modification of bone metabolism generated by ALL-B cells has been previously demonstrated [47], which explains the presence of vertebral fractures at diagnosis [50]. We did not observe significant differences in BAP levels, which could be explained by the fact that 40% of patients had elevated concentrations of BAP (≥75th percentile) at diagnosis, while 45% had elevated concentrations of BAP at remission. This is most likely compensatory to disorders of bone metabolism present in these patients, as high levels of BAP and its association with decreased lumbar BMD have been reported, especially in older women [51]. Additionally, our study population was largely composed of schoolchildren, and studies have reported the highest levels of BAP in this population [31,52].

Nevertheless, even though we detected changes in the RANKL level, OPG level, and RANKL/OPG ratio in the deficient and HR groups, Spearman correlation analysis of the bivariate BTM did not reveal a significant association between vitamin D concentration and corticoid dose. Similarly, Muggeo et al. [9] did not observe significant correlations between bone turnover marker levels and vitamin D levels. In contrast, Hablas et al. reported a negative correlation between circulating vitamin D and RANKL levels and a positive correlation with OPG levels in 60 ALL survivors. This was probably due to differences in sample size, population, and, mainly, the proportion of patients with >30 ng/mL vitamin D (30% vs. ~9% [two patients]).

The principal limitations of this study were the lack of a control group and the evaluation of BMD. However, our study used a larger sample than that of Solmaz et al. [34], providing good statistical power. In addition, we explored the changes in a cohort from diagnosis to complete remission according to 25(OH)D status, risk of relapse, and cumulative doses of corticosteroids. We analyzed the evolution of the same group of patients at diagnosis and remission, in contrast with other studies [9]. Another strength of this study was evaluating the changes in BTM and the RANKL/OPG ratio in a cohort of patients with ALL without any intervention in addition to early hematological treatment.

## 5. Conclusions

During remission, we observed an increase in RANKL and osteocalcin levels and a decrease in OPG levels. When relapse risk and vitamin D levels were considered, patients with an HR of relapse or vitamin D deficiency presented these changes. Therefore, the RANKL/OPG ratio could be a good marker of bone remodeling in patients with ALL. Hypovitaminosis D and increased RANKL/OPG may predispose such patients to the development of bone health disorders, such as osteopenia, osteoporosis, and increased bone fragility, in their adult lives. To better understand bone remodeling markers in children with leukemia, it is necessary to conduct future studies.

## Figures and Tables

**Table 1:** Characteristics at diagnosis of acute lymphoblastic leukemia in children.

Variable	All N = 33
Demographic	
Gender	
Male, n (%)	20 (61)
Female, n (%)	13 (39)
Age (y)	9.2 (4.0, 17.7)
Anthropometric and body composition	
Body weight (Kg)	33.4 (13.3, 99.6)
Height (m)	1.4 ± 0.3
BMI (Kg/m^2^)	17.1 (12.1, 32.5)
BMI (percentile)	54.8 (3.0, 100.0)
Eutrophic (BMI pc > 5 pc < 85) n (%)	21 (64)
Undernourished (BMI pc ≤ 5) n (%)	4 (12)
Overweight (BMI pc > 85) n (%)	4 (12)
Obese (BMI pc > 95) n (%)	4 (12)
BMI (Z-score)	0.2 ± 1.5
Lean body mass (Kg)	12.5 (3.8, 40.7)
Fat mass (Kg)	6.1 (1.5, 42.2)
Fat mass (%)	22.5 ± 10.1
Clinical parameters	
Classification	
High risk, n (%)	21 (64)
Intermediate risk, n (%)	3 (9)
Standard risk, n (%)	9 (27)
Immunophenotype	
Pre-B, n (%)	33 (100)
Leucocytes (miles/µL)	7.8 (0.9, 425.3)
Leucopenia, n (%)	13 (39)
Hemoglobin (g/DL)	9.2 ± 2.2
Anemia, n (%)	31 (94)
Platelets (miles/µL)	39.0 (10.0, 425.0)
Thrombocytopenia, n (%)	30 (91)
Neutrophiles (miles/µL)	0.6 (0.1, 5.9)
Neutropenia, n (%)	24 (73)
Bone metabolism markers	
RANKL (pg/mL)	65.5 (4.0, 343.0)
OPG (pg/mL)	463.9 (210.9, 2661.7)
RANKL/OPG ratio	0.14 (0.004, 1.06)
BAP (IU/L) *	102.3 (44.8, 371.9)
BAP ≥ 75th percentile, n (%)	8 (40)
Osteocalcin (pg/mL)	24,098.4 (8900.3, 113,640.9)
TRAP (pg/mL) *	0.3, (0.2, 1.1)
25(OH)D (ng/mL)	17.4 (5.7, 61.9)
Sufficiency, n (%)	3 (9)
Insufficiency, n (%)	10 (30)
Deficient, n (%)	20 (61)

Data are expressed as mean ± standard deviation, median (interquartile range), or number (percentage). Vitamin D classification: sufficiency (≥30 ng/mL), insufficiency (21–29 ng/mL), deficiency (<20 ng/mL). * Sample size analyzed n = 20. RANKL: receptor activator for nuclear factor kB ligand; OPG: osteoprotegerin; BAP: bone alkaline phosphatase; TRACP-5b: tartrate-resistant acid phosphatase-5b.

## Data Availability

The data presented in this study are available upon request from the corresponding author.
